# Maine Veterinary Medical Association

**Published:** 1897-02

**Authors:** W. L. West

**Affiliations:** Secretary


### MAINE VETERINARY MEDICAL ASSOCIATION.

THE semiannual meeting was called to order by President Russell at Bangor, January 14, 1897. On calling the roll Drs. Choate, Russell, West, Lord, Salley, Joly, Murch, Cleaves, and Dwinal were present. The reports of the secretary and treasurer were read and approved. Dr. L. Sherman Cleaves, of Bar Harbor, was elected to membership.

The following officers were elected for the ensuing year: President, Dr. F. L. Russell; Vice-President, Dr. A. L. Murch; Secretary, Dr. W. L. West; Treasurer, Dr. A. Joly.

President Russell appointed the following Executive Committee: Dr. Huntington, Portland; Dr. Murch, Bangor; Dr. Salley, Skowhegan.

Dr. West moved that the Association give certificates of health to owners of herds tested and found healthy. After a very spirited discussion by Drs. Russell, Lord, Murch, Salley, Joly, Cleaves, and West, the motion was withdrawn.

It was voted to send five dollars to the Committee of the Pasteur Memorial Fund.

Dr. Joly moved that the Association ask the Legislature to amend the Public Law of 1887 for the Cattle Commission to consist of five instead of three members as at present; after a very free discussion, motion lost.

On motion of Dr. Joly, a committee of three was appointed to confer with the State Boards of Agriculture and Health in regard to the State Board of Health taking charge of contagious diseases. Dr. Russell appointed the following committee to confer with State Boards: Drs. West, Joly, and Lord.

Dr. Choate moved that the sentiments of the Association in regard to the Army Veterinary Bill be sent to Speaker Reed; the motion was carried, and the secretary instructed to attend to this matter.

On motion, voted to reconsider the action of the Association in regard to presenting the bill to the Legislature as in 1894.

On motion, voted that the Association pay the expenses of the committee to meet the State Boards.

Voted to submit to the Maine Legislature the bill which will be introduced into the Colorado Legislature. On motion, voted that a committee of three be appointed to look after the interests of the bill and see that it is properly presented to the Legislature. Dr. Choate, Dr. Salley, and Dr. Joly were appointed Committee on Legislation.

On motion, voted that a notice of amendment to the By-laws of the Association, Art. III., section 1, be made to read, "the meeting of this Association shall be held once in three months."

Voted to issue a call for a special meeting at Hotel, North Augusta, February 15, 1897, for the purpose of action in regard to Testing Cattle and Board of Health Acts.

Voted to adjourn.

W. L. WEST,
Secretary.

